# Novel Strategies for the Formulation of Poorly Water-Soluble Drug Substances by Different Physical Modification Strategies with a Focus on Peroral Applications

**DOI:** 10.3390/ph18081089

**Published:** 2025-07-23

**Authors:** Julian Quodbach, Eduard Preis, Frank Karkossa, Judith Winck, Jan Henrik Finke, Denise Steiner

**Affiliations:** 1 Division of Pharmaceutics, Utrecht Institute of Pharmaceutical Sciences, Utrecht University, Universiteitsweg 99, 3484 CG Utrecht, The Netherlands; j.h.j.quodbach@uu.nl; 2 Department of Pharmacy, Institute of Pharmaceutics and Biopharmaceutics, Philipps-Universität Marburg, Robert-Koch-Str. 4, 35037 Marburg, Germany; eduard.preis@pharmazie.uni-marburg.de; 3 Department of Pharmacy, Institute of Biopharmaceutics and Pharmaceutical Technology, Center of Drug Absorption and Transport (C_DAT), University of Greifswald, Felix-Hausdorff-Straße 3, 17489 Greifswald, Germany; 4 Laboratory of Solids Process Engineering, TU Dortmund University, Emil-Figge-Str. 68, 44227 Dortmund, Germany; judith.winck@tu-dortmund.de; 5 Institute for Particle Technology, Technische Universität Braunschweig, Volkmaroder Str. 4, 38104 Braunschweig, Germany; jan.finke@tu-braunschweig.de; 6 Center of Pharmaceutical Engineering, Technische Universität Braunschweig, Franz-Liszt-Str. 35A, 38106 Braunschweig, Germany; 7 Fraunhofer Institute for Surface Engineering and Thin Films IST, Riedekamp 2, 38108 Braunschweig, Germany; 8 Institute of Pharmaceutical Technology and Biopharmaceutics, University of Muenster, Corrensstraße 48, 48149 Muenster, Germany

**Keywords:** drug nanoparticles, solid dispersions, lipid-based formulations, physical modification, bioavailability, drug carrier systems, drug delivery systems

## Abstract

The number of newly developed substances with poor water solubility continually increases. Therefore, specialized formulation strategies are required to overcome the low bioavailability often associated with this property. This review provides an overview of novel physical modification strategies discussed in the literature over the past decades and focuses on oral dosage forms. A distinction is made between ‘brick-dust’ molecules, which are characterized by high melting points due to the solid-state properties of the substances, and ‘grease-ball’ molecules with high lipophilicity. In general, the discussed strategies are divided into the following three main categories: drug nanoparticles, solid dispersions, and lipid-based formulations.

## 1. Introduction

High bioavailability of medicinal products is often a prerequisite for effective drug therapy. Among other critical parameters, drugs’ solubility in relevant fluids of the human body (e.g., gastrointestinal fluids in the case of an orally administered dosage form) and their ability to cross the biological barrier [1] are two key determinants of their bioavailability. Formulation scientists worldwide are continuously working on new ways to ensure that high percentages of a drug can be absorbed by an organism. However, the differing physicochemical properties of drugs can cause considerable challenges. One of the most prominent challenges is poor solubility in water. As many newly discovered drug candidates are poorly water soluble [2], overcoming this challenge becomes increasingly important, necessitating various strategies to solve this limitation.

Poorly water-soluble drugs are classified in the BCS (Biopharmaceutics Classification System) classes II or IV, which means that a single dose of the drug is not fully soluble in 250 mL of aqueous liquid [3,4]. Despite sharing this characteristic, further physicochemical properties of the substances in these classes can vary significantly. According to the General Solubility Equation (GSE) for organic nonelectrolytes, developed by Yalkowsky and Valvani [5], the following two key factors generally influence the solubility of a substance: the melting point (T_m_) and the octanol-water partition coefficient (logP) [6]. In order to enable rough distinction among poorly water-soluble drugs, they are differentiated according to the component that mainly limits their solubility. High melting points indicate the limited solubility of a substance due to solid-state properties and are referred to as ‘brick-dust’ molecules. When solubility is mainly limited by solvation, indicated by high logP values, they are called ‘grease-ball’ molecules [7]. Dependent on these factors, a rough estimation of a suitable formulation strategy can be derived. Lipophilic compounds (‘grease-ball’ molecules), characterized by high logP values, are often formulated in lipid-containing formulations, while high melting points indicate hydrophobic compounds, which are usually formulated in a modified solid state [4].

## 2. Research Strategy

This review provides an overview of various physical modification strategies that can be used to improve the bioavailability of drugs, with a focus on oral dosage forms. Divided into three main strategies—drug nanoparticles, solid dispersions, and lipid-based formulations—each section briefly introduces the relevant technology. A literature search was conducted focusing on technologies related to the three main strategies identified in this field. Leading studies on these strategies and novel approaches published over the last decades were then selected to provide an overview of current research activities in this field and are summarized in the following review article.

## 3. Preparation of Drug Nanoparticles

A decreasing drug particle size significantly increases the total surface area, which enhances the dissolution rate, as described by the Noyes–Whitney and Nernst–Brunner equations [8,9]. Furthermore, it is discussed whether the surface curvature of nanoparticles could lead to an increase in the saturation solubility, as described by Kelvin [10] and Ostwald–Freundlich equations [11], which would also lead to a faster dissolution of a drug. Physical approaches applied for the preparation of nanoparticles are mainly divided into top-down methods, such as nanomilling, and bottom-up methods, like precipitation. Both mentioned technologies are the most prominent representatives of these methods and are discussed in detail below.

The term ‘nanoparticle’ or ‘nanoscale’ is, in general, used differently in the pharmaceutical context than is intended with the original definition. According to ISO/TS 80004-1:2023, a nanoparticle is a ‘nano-object with all external dimensions in the nanoscale’, meaning between 1 and 100 nm [12]. When reviewing the pharmaceutical literature, the terms nanocrystals or nanoparticles are often used somewhat differently, namely, for particles in the size range between (sometimes 1 nm but more commonly) 100 and 1000 nm (e.g., [13,14,15]). However, studies indicate that targeting drug particles with sizes below approx. 300 nm can significantly enhance the bioavailability of poorly water-soluble drugs [16].

### 3.1. Nanomilling of Drug Particles

In the top-down approach to preparing drug nanoparticles, wet media milling is primarily used in the pharmaceutical industry [17]. Other methods, such as high-pressure homogenization, can also be found in the literature [13], but they are not discussed in this review. The first experiments on nanomilling were carried out by Liversidge and co-workers in 1995. They prepared nanoparticles of the drugs naproxen [18] and danazol [19] with mean particle sizes of 169 nm and 270 nm, respectively, and showed a significant increase in bioavailability for both drugs in in vivo studies in rats [18] and beagle dogs [19]. In subsequent years, numerous drugs were milled using various technologies. The following provides an overview of the technologies applied, challenges encountered, and current studies focusing on processing these nanosuspensions into solid forms.

#### 3.1.1. Milling Technologies

In principle, wet media milling describes the mechanical stressing of product particles dispersed in a liquid with grinding beads. The grinding beads can consist of various materials, such as glass or ceramics (e.g., aluminum oxide and yttrium-stabilized zirconium oxide). In some studies, organic milling beads from highly cross-linked polystyrene [20] or frozen water droplets, which melt during the milling process, were used to reduce inorganic product contamination [21]. Depending on the type of mill, the beads are set in motion in different ways. The first experiments by Liversidge et al. were carried out in a roller mill, a cylindrical vessel filled with the suspension and milling beads, which was rotated horizontally for 120 h, with the forces applied being basically the weight forces of the grinding beads [18,19]. Mills such as stirred media mills, in which the beads are set in motion by a rotating rotor, are more suitable, resulting in a much higher energy density and a significant reduction in grinding time. It was shown that particle sizes below 200 nm can be achieved in approx. 60 to 120 min, depending on the formulation [20,22], whereby various mill settings, such as the batch or recirculation mode, can be chosen. Planetary ball mills are operated in the batch mode and use centrifugal forces to move the beads in a closed milling beaker. The milling beakers rotate around their own axis while they are fixed to a disk that rotates around the central axis. This design results in high centrifugal forces, and particles can be stressed in beakers with a volume of up to 1 L [23]. Juhnke et al. introduced a newly designed beaker holder that enables the fixation of up to 24 zirconium dioxide milling beakers with a 0.05 to 1.0 mL individual chamber volume in four milling spaces in order to minimize material consumption and enable formulation screenings at an early stage of formulation development when only limited amounts of the drug are available [24]. Recent studies have shown that screening experiments can also be performed in a dual centrifuge in 2 mL polypropylene screw-cap vials equipped with grinding beads, enabling batch sizes between 10 mg and 1 g. The dual centrifuge differs from the planetary ball mill in that it orients the vials horizontally, which significantly reduces the processing time [25]. In addition, dual centrifugation shows advantages when it comes to narrow size distributions and the processing of formulations with drug concentrations of up to 40%, while experiments with a planetary ball mill showed limitations due to the increased viscosities of these formulations [26]. Upscaling experiments from a dual centrifuge to larger-scale stirred media mills were carried out successfully [27].

In the majority of studies found in the literature to date, water has been utilized as the continuous phase for particle milling. Only a few studies used other liquids such as ethanol [28] or oil [29]. However, all of these studies have in common that they were conducted at room temperature or even actively cooled during processing to prevent heating of the formulation due to mechanical stressing during processing. Igreja et al. chose a different approach and milled griseofulvin particles in a melt containing the matrix material xylitol to stabilize the milled particles directly in a matrix that turns solid at room temperature to improve long-term stability. At milling temperatures of 120 °C, the drug was milled in a custom-built annular gap mill and achieved particle sizes below 300 nm [30,31].

#### 3.1.2. Challenges in the Preparation of Nanoparticles Using Milling

Although milling poorly water-soluble drugs has been shown to improve their bioavailability significantly, there are also challenges that must be addressed during formulation development. A common issue when working with nanoparticles is the thermodynamic instability caused by the large surface area of the particles. To prevent agglomeration of the particles, surface-active additives are added to reduce the free energy of the system and ensure colloidal formulation stability. This can be achieved either by electrostatic repulsion between the particles through the addition of charged or ionic surfactants or by steric repulsion through the addition of non-ionic polymers. A combination of both methods is also possible (electrosteric stabilization) and often used to stabilize drug particles [15,32,33]. Until now, the selection of suitable additives for particle stabilization has mainly been empirical. Recent studies have shown that the Hansen solubility parameters (HSP) could be helpful in evaluating the stabilization capacities of polymeric additives [34,35]. In addition to particle agglomeration, crystal growth phenomena have been observed during wet media milling of particles. These recrystallization and ripening effects are presumably caused by mechanically induced dissolution effects and increased dissolution of the particles after milling [28,32,36,37].

The use of inorganic grinding beads to mechanically stress drug particles can lead to wear and, thus, product contamination, which is a critical concern in pharmaceutical formulations. Even when using high-quality grinding beads, the generation of wear particles cannot be eliminated entirely; rather, this can be reduced by optimizing the grinding process. Various studies have shown that the process parameters, for example, of a stirred media mill, have a significant influence on wear generation [28,38,39]. Recently, Flach et al. found that wear particles are present in nanosuspensions and actively interact with the (nano)particles of a product. They showed that electrosteric stabilization of a suspension can lead to the formation of heteroagglomerates between the product and wear particles due to electrostatic attraction [40]. However, they also observed that product particles can act as a kind of protective shield around the milling beads, protecting them from abrasion. In this experimental setup, the particles are milled in an agglomerated state and dispersed by adding a subsequent stabilizer after the milling process [41].

#### 3.1.3. Processing of Nanosuspension into Solid Form

Drug nanosuspensions are generally unstable systems. Although they can be properly stabilized during the milling process, they often have issues of physical stability in addition to chemical degradation and microbial contamination, which makes achieving long storage times for the formulations difficult. To overcome these stability issues, the nanosuspensions can be further processed into solid forms by various drying methods. Common to all strategies is that they aim to maintain particle stability during further processing in solid form to not affect the improved dissolution rate of the drugs. Recent studies on these different technologies are presented in the following.

During spray drying, a nanosuspension is atomized into a heated gas stream where the liquid evaporates. While thermal stress could have an impact on the redispersibility properties of the nanoparticles [42,43,44], studies have also successfully prepared nanoparticle-containing spray-dried powders with low inlet temperatures, e.g., 100 °C [45]. To enable the easy redispersion of nanoparticles from dry powders, they are often processed with matrices such as sugars, sugar alcohols, or polymers. These (water-soluble) additives are added to the nanoparticle suspension before spray drying to enable the embedding of the nanoparticles during the drying process [43,45,46,47]. Czyz et al. used various sugars and sugar alcohols as additives in the drying process of naproxen and itraconazole nanoparticles. They showed that if good particle redispersibility is to be obtained, the maximum possible drug content in the dried powders is temperature-dependent and that the interaction between the drug particles and the matrix material plays an important role. In addition, they found that with a decreasing particle size, high drug loads become more challenging, and, therefore, an optimum between high amounts of drugs and high dissolution rates needs to be found [48].

Freeze drying is another technology that forms dry nanosuspensions. It is advantageous when heat-sensitive drugs are processed or highly porous structures are to be achieved, e.g., to obtain rapid disintegration in water. Good redispersibility of nanoparticles was also demonstrated with this technology, although further processing of the dried powders could pose a challenge due to the formation of a so-called ‘cake’. In order to prepare spheres with good flowability, the technology was recently further developed with spray–freeze drying [49]. Pellets are formed from a nanosuspension by dripping the formulation into liquid nitrogen before freeze drying. Stable pellets were achieved by adding matrix materials, such as hydroxypropyl cellulose SSL (HPC SSL) [50], polyvinylpyrrolidone (PVP) [51], and sucrose laurate or lactose laurate [52], to obtain well redispersible and low-friable dried pellets [50].

Fluidized bed granulation also yields powders with good flowability. Nanosuspensions were used as granulation liquid and to form a thin soluble shell on carrier particles [53]. Several formulation parameters were determined to influence the dispersibility of nanoparticles in water. First, it was shown that the solubility of the unloaded carrier material of sugar or sugar alcohols influences the redispersibility of the nanoparticles [54,55]. When different carrier material particle sizes were investigated, the results showed that faster dissolution rates were achieved when carriers with smaller particle sizes were used [53]. In addition, the amount of polymer in the nanosuspension was identified as another important formulation parameter. Higher amounts of polymeric additives, such as hydroxypropyl methylcellulose (HPMC) [53,54,55], PVP [56], or vinylpyrrolidone-vinyl acetate copolymer (PVA/VA 64) [54,55], indicated an improved redispersibility of the nanoparticles. Wewers et al. attributed this to the greater distance between the drug nanoparticles in the shell around the carrier particles [54]. Other studies that performed in vivo experiments showed that no differences could be detected between the liquid nanosuspensions and the dried powders, regardless of the spray modes used (top or bottom spray) [57,58], although differences in the spray mode were detected when the powders were analyzed with an in vitro redispersion method [57].

Another, more innovative strategy to maintain nanoparticle size is the embedding of particles in a film-forming matrix. These are dried and form, for example, orodispersible films (ODFs). The films disintegrate and dissolve directly in the mouth so that drug nanoparticles can be swallowed with saliva [59]. Polymers used to embed nanoparticles include HPMC, polyvinyl alcohol (PVA), gelatin, maltodextrins, and starches [60,61]. Nanoparticle loadings of up to 50 wt.% were realized, with only slight increases in particle sizes after the redispersion of the films [62,63]. In addition, Steiner et al. developed a method to prepare a nanoparticle-containing solvent casting mass directly in a stirred media mill to obtain high particle loadings in ODFs and reduce additional preparation steps. Directly after particle milling, they added HPMC powder to the storage vessel of the running stirred media mill and took advantage of the high shear forces of the grinding media to dissolve and homogenize the polymer [64]. Several milled drug nanoparticles have already been embedded in ODFs, such as itraconazole [65], loratadine [66], and fenofibrate or naproxen [67].

Recently, drug-free ODFs have been used as a template for 2D printing of nanoparticular suspensions. Various ODF-like templates can be used, as follows: films with smooth surfaces or more porous films prepared from HPMC by freeze drying [68], or solvent casting of an ethanol HPMC suspension—so-called SOFTs (structured orodispersible film templates) [69]. Milled nanoparticulate drugs such as indomethacin and itraconazole [70], as well as naproxen [71], have already successfully been used in printing. Inks contain different additives such as surfactants to stabilize the particles in the liquid formulation [72] and preserve the particle sizes after ink dries on a template [71].

### 3.2. Precipitation of Drug Nanoparticles

In contrast to top-down methods, such as milling or high-pressure homogenization, bottom-up methods, which start from molecular solutions of drug substances, are also studied for the preparation of drug nanosuspensions. General overviews and descriptions of the variety of precipitation methods studied are presented elsewhere [73,74,75]. Overall, liquid antisolvent precipitation and so-called flash precipitation in microsystems, which apply very short mixing times, are the most frequently used methods. In these processes, a poorly soluble drug is dissolved in a solvent with sufficient solubility for the drug. This solvent drug solution is then injected into an antisolvent, with which the solvent is miscible. Due to the high supersaturation and applied rapid mixing kinetics, a high number of nuclei will be produced to form drug nanoparticles. In this context, solvent properties influence the precipitation process and the resulting drug’s nanoparticle size distribution [76]. As defined, continuous mixing is mostly applied in low-flow-rate microfluidics, and production rates usually remain small. High-pressure antisolvent precipitation up to 230 mL/min was studied as a novel approach [77], in addition to melt precipitation methods at elevated temperature [78], which is, indeed, only suitable for drugs with very low melting points (such as ibuprofen). For the efficient production of drug nanosuspensions and to disperse and stabilize precipitated drug nanoparticles, combined methods of precipitation and high-pressure homogenization, as well as other top-down methods, have been investigated [79,80].

The drawbacks of such bottom-up precipitation methods are the necessity of finding suitable and water-miscible solvents, dealing with solvent residuals in the product, and the specific challenge of scaling-up such processes to an industrial scale [81]. Due to these drawbacks, precipitation methods are less prominent in industrial applications for the production of drug nanosuspensions.

## 4. Solid Dispersions

The formation of solid dispersions is another physical formulation strategy to improve drug solubility. The principal idea of solid dispersions was first described by Sekiguchi and Obi in 1961 [82]. While investigating eutectic mixtures of the poorly soluble drug sulfathiazole with different excipients, they developed the concept of finely dispersed drug particles within easily soluble compounds to improve drug dissolution. This initial description has been expanded to accommodate further developments, and, to date, three different classes of solid dispersions have been considered [83], as follows:

(1) Eutectic mixtures;
(2) Solid solutions;
(3) Crystalline dispersions.

**Figure 1:**
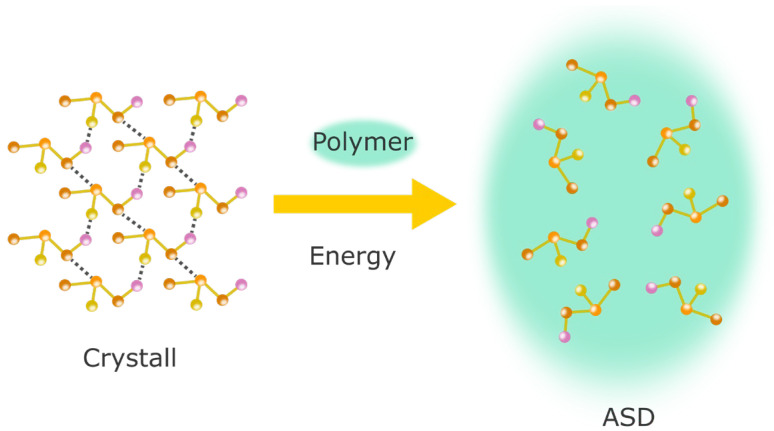
Simplified structure of an ASD.

A eutectic mixture is a mixture of two compounds in a specific ratio that shows a single melting point below the melting points of the individual compounds. Solid solutions can be further differentiated into substitutional solid solutions, where a solute molecule replaces a solvent molecule; interstitial solid solutions, where solute molecules are distributed within the interstices; and amorphous solid solutions or amorphous solid dispersions (ASDs), where a drug is molecularly dissolved in an amorphous carrier. Crystalline dispersions follow the description of Sekiguchi and Obi, who described the presence of crystalline drugs in a given carrier system. Generally, it has to be said that the term ASD is commonly used for solid solutions and solid dispersions, denoting the formulation principle (drug + polymer) rather than the phase behavior (Figure 1). The mechanisms by which solid dispersions improve drug solubility depend on the type of dispersion formed and include the elimination of crystalline lattice energy by conversion to the amorphous state, a reduction in the particle size, improved wetting, and improved solubilization [4]. Another classification system for solid dispersions proposes the existence of four distinct generations [84]. The first generation includes crystalline and the second generation amorphous carriers. The third generation improves on the second generation by including carriers with surface activity or emulsifying properties. Fourth-generation solid dispersions not only exhibit improved solubility but also show extended release. As classification by generation is comparably diffuse, within the scope of this review, the traditional nomenclature listed before is considered.

In the following sections, the fundamentals and recent developments related to the different approaches to manufacturing, processing, and stabilizing solid dispersions are discussed. Separate sub-sections are dedicated to ASDs, crystalline dispersions, mesoporous systems, and aerogels.

### 4.1. Amorphous Solid Dispersions (ASDs)

Among the various approaches to enhancing dissolution kinetics and solubility, ASDs hold a special position, as they combine the benefits (and unfortunately disadvantages) of amorphous materials with improved wettability and solubilization utilizing a soluble matrix.

**Figure 2:**
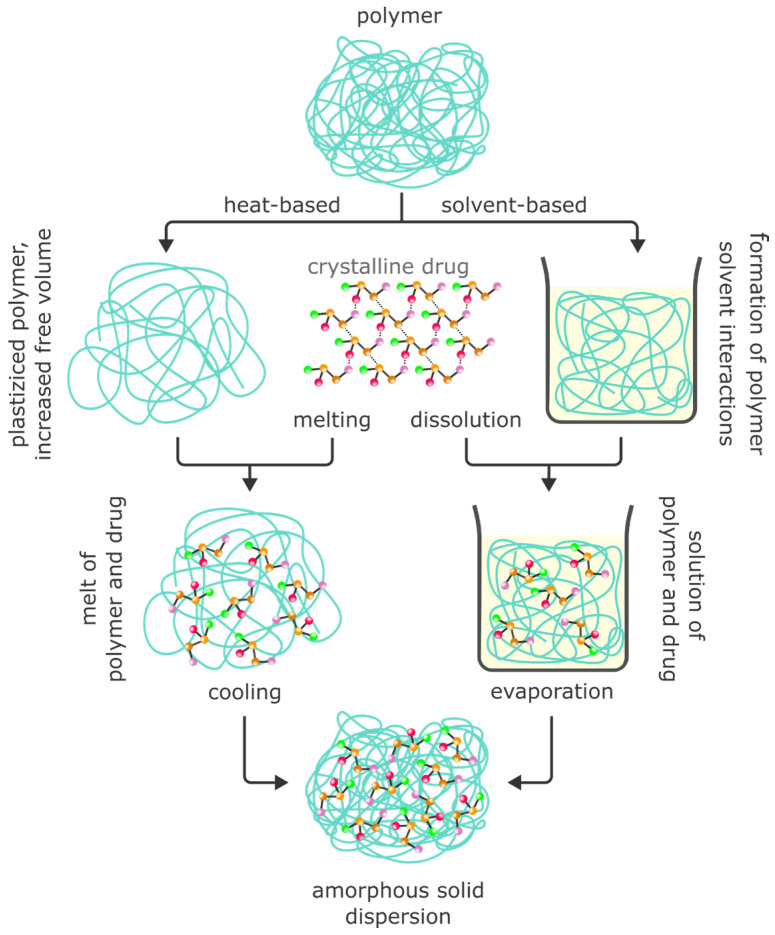
Different manufacturing technologies used for the preparation of ASDs, according to [85].

Different technologies have been developed for manufacturing ASDs, and recent developments and novel approaches are presented. The following section first covers the theoretical background of preparing ASDs and then examines the methods, including spray drying, hot-melt extrusion, and electrospinning, used to prepare ASDs. An overview of the discussed technologies is presented in Figure 2.

#### 4.1.1. Theoretical Background to the Preparation of ASDs

As the name implies, the amorphous states of the drug and carrier play crucial roles in the performance of ASDs. The major application cases for solid dispersions are the improvements in the drug dissolution rate and drug solubility, which are among the most important critical quality attributes of dosage forms. For a crystalline drug, three steps have to be passed for the drug to dissolve in a given solvent [86]. In an endothermic reaction, the crystalline lattice has to be disrupted. To host the free drug molecules that detach from the crystalline lattice in the solvent, ‘cavities’ must be created in the solvent via an endothermic reaction called cavitation. In the last step, the drug molecules are hydrated in the cavities via an exothermic reaction. These processes occur in parallel, and the full dissolution process can be overall endo- or exothermic depending on the energy contributions of the endo- and exothermic steps. Following this description, two properties—the lattice energy of the crystalline drug and the hydrophilicity of the drug molecules—govern the dissolution of the crystalline drug in a given medium. Since drug molecules in ASDs are molecularly dispersed, no lattice energy has to be overcome, and dissolution is strongly facilitated.

**Figure 3:**
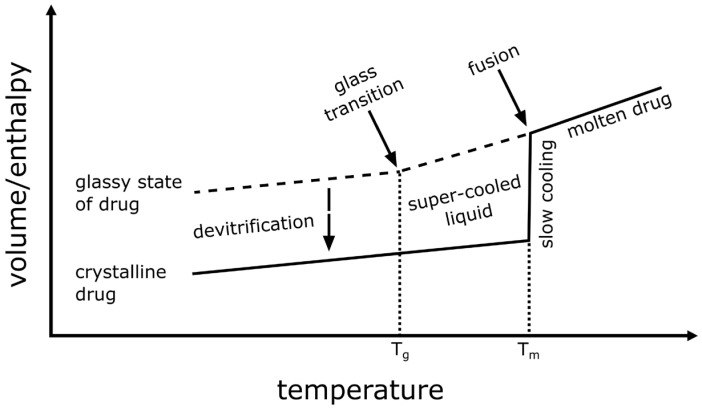
Enthalpy and volume of crystalline drugs in various states as a function of the temperature, according to [83].

To convert a drug substance to the amorphous state, sufficient energy must be put into the system to break up the complete crystalline lattice. In Figure 3, the volume and enthalpy of a drug are plotted against temperature. Starting with a crystalline drug, the volume and enthalpy increase linearly during heating until T_m_ is reached. At T_m_, the thermal energy surpasses the lattice energy, and the intermolecular bonds are broken. This is accompanied by strong increases in the volume and enthalpy as the drug molecules now have greater mobility. With further heating, the volume and enthalpy continue to increase linearly, albeit with a higher slope. Upon slow cooling, this process chain is fully reversable. At T_m_, the molecules fuse and recrystallization takes place. If the material is quench-cooled, however, the kinetic processes of fusion and recrystallization are prevented, and the molecular mobility is reduced sufficiently to stabilize the amorphous state in a so-called super-cooled liquid. During quench-cooling while still above the glass transition temperature, T_g_, the volume and enthalpy decrease linearly. Upon passing T_g_, the slope of the volume and enthalpy decreases, which indicates a further reduction in the molecular mobility. The solid amorphous state is, at all temperatures, energetically less favorable and metastable, meaning that recrystallization can occur spontaneously.

**Figure 4:**
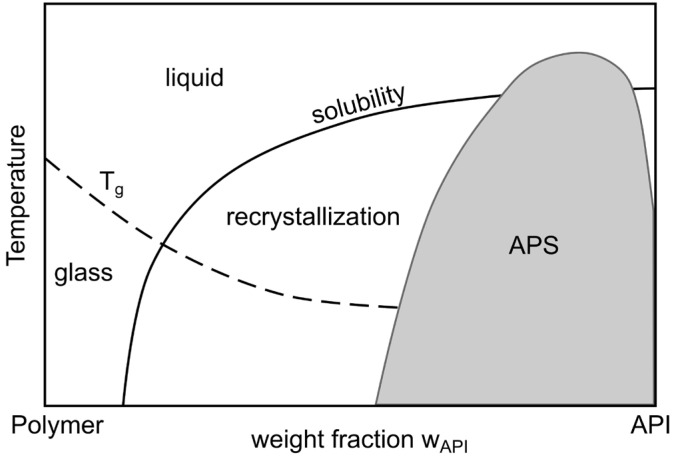
Phase diagram of a drug/polymer formulation. The T_g_ line is shown as a dashed black line, and the solubility line as a solid black line. The gray area denotes the amorphous–amorphous phase region. Below the solubility line, recrystallization occurs, according to [87].

In ASDs, drug molecules are dispersed in amorphous matrix materials, commonly polymers, to increase the stability of the drug’s amorphous state. In Figure 4, a schematic phase diagram of a binary ASD formulation, comprising a drug and polymer, is shown. At weight fractions above the solubility line and below the T_g_ line, ASDs are stable solids. Above both—the solubility and T_g_ lines—the system is in a liquid state. Below the solubility line, the mixture is metastable, and recrystallization may occur. However, the amorphous state of the drug is kinetically stabilized via entrapment in the polymeric matrix. The time to recrystallization greatly depends on the diffusion coefficient of the drug molecules and their interaction potential. Below the T_g_, the drug diffusion coefficient is much lower than if above the T_g_, and the ASD is kinetically stabilized. On the other hand, a high molecular affinity of the drug molecules toward each other favors faster recrystallization. At high weight fractions of the drug, amorphous–amorphous phase separation will take place.

The drug–polymer phase behavior is a critical factor in formulation design. A carrier polymer that enables the highest possible drug solubility should be selected, ideally resulting in a single-phase system that remains stable throughout storage. Different models have been developed over time to calculate drug-in-polymer solubility. Recently, a new model that predicts drug-in-polymer solubility using an easily quantifiable thermodynamic property, the enthalpy of melting, was proposed [88]. A free and open-source software application, published alongside this model, simplifies its experimental and practical implementation [89]. Another approach to achieve drug loadings above the thermodynamic solution limit is kinetic stabilization. In such cases, a drug is not fully soluble in the polymer at storage temperature, but stability is maintained over a defined storage period due to kinetically hindered molecular mobility (reduced diffusion coefficient). A high T_g_ of the polymer is advantageous in this context, allowing the ASD to be stored well below its T_g_. In general, higher drug loading compromises the physical stability, as the T_g_ of the ASD decreases with an increase in drug content. Therefore, an optimal balance must be sought between drug loading and sufficient storage stability for the intended application.

#### 4.1.2. Spray Drying of Protein-Based ASDs

The spray drying of ASDs is a process in which a drug solution with stabilizing excipients rapidly dries from a fine spray at elevated temperatures. Water and organic solvents can be used to solubilize the drug and other formulation ingredients. Despite a high gas temperature, heat-sensitive drugs, including biologics, can be processed, as the drying rate is so high that most of the heat dissipates with the solvent vapor. Spray drying is an energy-intensive but highly versatile process, about which multiple extensive reviews have been written [90,91]. Because of its versatility, spray drying is frequently employed industrially and is of high interest to researchers. Recently, the following novel excipient class for the stabilization of ASDs in spray drying was introduced: proteins.

Initial studies that used ball milling to prepare ASDs based on whey protein isolate found strongly increased T_g_ values, faster dissolution rates, and high stability of the prepared ASDs [92]. In a later study, also utilizing ball milling, different isolated whey proteins were compared, and it was shown that β-lactoglobulin (BLG), the major component in whey protein isolate, outperformed the other dominant isolate components, α-lactalbumin and casein glycomacropeptides [93]. In a comparison with a common stabilizing polymer, PVP, BLG showed a higher amorphous drug-loading capacity and faster drug release [94]. Spray drying was used to compare ASDs based on BLG with formulations containing hydroxypropyl methylcellulose acetate succinate (HPMC AS) and Eudragit^®^ L containing 50% (*w*/*w*) of the model drug rifaximin [95]. While rifaximin was fully amorphized in all ASDs, dissolution in media at pH 1.2 and 4.5, as well as FaSSIF-V2 (fasted state simulated intestinal fluid V2), was faster for the BLG-based ASDs, which also showed the highest T_g_, indicating improved storage stability. In simulations, the main stabilization mechanisms in BLG-based ASDs were identified to be steric confinement and hydrogen bonding with lesser contributions by inter-drug hydrogen bond networks, ionic interactions, and increased T_g_ values [96]. To date, BLG has been tested with multiple model drugs, showing promising results. However, there is still a lack of research on the widespread applicability of BLG, other proteins and protein classes, and manufacturing technologies, in addition to ball milling and spray drying.

#### 4.1.3. Hot-Melt Extrusion

Hot-melt extrusion (HME) is a versatile thermal manufacturing process and was used for the first time in a pharmaceutical context in 1971 [97]. In HME, powdered material is transported in a heated barrel along one or multiple screws. The material is melted or plasticized along the screw(s) either via the energy input from the heated barrel and shear stress caused by the rotating screw or only via the shear stress (autogenic extrusion mode) [98]. The melted drug is intimately mixed with the plasticized polymeric carrier to form an ASD. After extrusion through a die, the material cools down to room or storage temperature. To improve the dispersive and distributive mixing in the process, kneading and mixing zones are usually incorporated in the screw design. In pharmaceutical manufacturing, co-rotating twin-screw extruders are preferred over single-screw extruders because of their increased flexibility, enhanced mixing capabilities, better melt temperature control, and reduced residence time [99]. HME has been widely applied in the preparation of ASDs because it can be considered a near-optimal thermal process to mix poorly soluble drugs with viscous polymer carriers [100]. While HME is a highly capable process for improving drug solubility, the downsides include the demand for good powder flowability and feedability to ensure reproducible manufacturing and the use of high process temperatures, which may limit HME to thermostable drugs [101]. In the following, novel HME approaches to address the process shortcomings or to expand the applicability of the technique are presented and discussed:

a. HME with carbon dioxide

As stated above, one of the major challenges of ASD manufacturing via HME is the high process temperature required to plasticize the polymer and dissolve the drug in a molecularly dispersed form [102,103]. The thermolabile drug can subsequently degrade and, in addition to reduced effectiveness, form toxic decomposition products [83,104].

A promising strategy to reduce the required temperatures during polymer extrusion is to use carbon dioxide (CO_2_) as a plasticizing agent [105,106]. By diffusing into the polymer, CO_2_ increases the free volume between the polymer chains, thereby leading to plasticization at lower temperatures [107]. CO_2_ is particularly suitable as a plasticizing agent, as its chemical structure facilitates a high solubility in most polymers [108]. Additionally, CO_2_ is an ideal candidate for pharmaceutical applications due to its inert, non-toxic nature, and it can be removed completely from the final product through decompression, leaving no harmful residues [109].

CO_2_-assisted extrusion of ASDs showed less degradation of the products, compared with conventional HME processes [110], and faster drug release [111,112], which can be explained by the increased surface area of the extrudates [113,114]. The porous structure also improved the downstream processing of the extrudates. For example, during grinding, higher efficiency and comparatively smaller particle sizes can be achieved with a lower energy input [115,116,117,118]. Furthermore, tablets manufactured from porous extrudates exhibited improved mechanical properties, including greater hardness, higher tensile strength, and reduced friability [116,119]. Stability tests have shown that products processed with CO_2_ exhibit comparable stability to those processed without it [117]. Although these application studies highlight the potential of optimizing thermal ASD processing through utilization of CO_2_ as a processing agent, there is a lack of fundamental knowledge concerning the effect of pressurized CO_2_ on the interaction and phase behavior of the polymer and drug. In a recent study, the influence of CO_2_ on the dissolution kinetics and phase behavior of ASDs was investigated using high-pressure differential scanning calorimetry. Some of the studied formulations showed phase separation under CO_2_ loading, which must be considered in the design process [120].

b. HME and 3D printing using Fused Deposition Modeling (FDM)

HME is a fairly common industrial process in the manufacturing of commercial volumes. Because of its continuous nature and the required time to reach process equilibrium, HME is inherently not suitable for the economic preparation of small or even individualized batches. However, small or even individualized batches and medicines are necessary to fulfill the demands of personalized medicine or to supply individualized medication to pediatric, geriatric, or veterinary patients that cannot be adequately treated with existing medication. Personalized medicine encompasses the concept of utilizing ‘individuals’ phenotypes and genotypes […] for tailoring the right therapeutic strategy for the right person at the right time […]’ [121]. The advent of 3D printing technologies, specifically the fused-deposition modeling (FDM) technique, has introduced a new technology to HME downstream processing. FDM belongs to extrusion-based 3D printing technologies and utilizes continuous filaments as feedstock material, which are produced via HME. These filaments are fed into a heated print head where they are plasticized and deposited on a print bed. The print bed and print head contain actuators that enable movements in the x-, y-, and z-directions of the print head relative to the print bed. This way, 3D structures can be constructed layer-wise from the bottom up. Filaments contain at least one drug and a thermoplastic (polymeric) carrier but can contain further additives to improve the printing performance [122].

**Figure 5:**
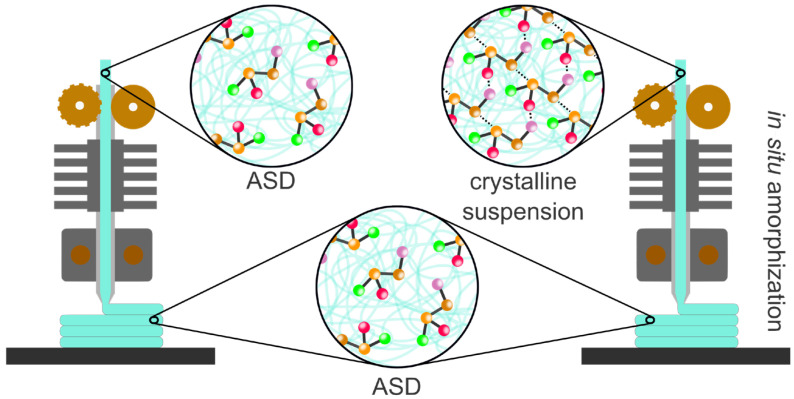
Approaches to the preparation of ASDs from filaments made from amorphous formulations or crystalline suspensions using FDM.

As FDM is a downstream processing technology for HME, printed medications can show the same benefits as hot-melt extruded materials. Specifically, FDM can utilize ASD formulations for printing (Figure 5). The ability to increase the drug solubility of BCS class II and IV drugs by processing ASDs into 3D printed forms has been demonstrated in multiple studies [123,124]. Anaya et al. developed a polypill containing nifedipine, simvastatine, and gliclazide, in which all drugs were fully amorphized in the HPMC AS-based matrix [125]. Parulski et al. extruded four formulations containing 25% itraconazole as the model drug [126]. In a 52-week stability study, no recrystallization was found for the printed dosage forms containing only HPMC as the polymer. As mentioned above, FDM requires a second heating step for the actual printing process, which can be leveraged. This additional heating step, which is usually performed at temperatures 20–40 °C above the extrusion temperature to plasticize the material within the short residence time in the print head, can be used to amorphize the drug. Buzukgoz et al. demonstrated this approach for griseofulvin and Hoffmann et al. for escitalopram oxalate [127,128]. This approach mitigates the risk of drug recrystallization in the filament during storage prior to printing. Generally, it can be assumed that every ASD that has the mechanical properties to be printable can be printed in individualized medication and that development approaches and analytical technologies used for ASD development in HME can be directly transferred to FDM. No studies have reported reduced stability after FDM printing.

Unfortunately, FDM also introduces additional process risks. The second heating step to temperatures above the extrusion temperature increases the possibility of drug degradation, which has been investigated in detail for the peptidomimetic drug enalapril maleate [129]. The transition from traditional HME processes, where extrudates are pelletized or milled, to the preparation of continuous filaments also increases process and product demands. Critically, inhomogeneities in the diameter of the extrudate that were previously irrelevant, as the material was homogenized via milling or pelletization and followed by mixing, now became a critical quality attribute. They directly translate to weight and dosage inconsistencies in printed dosage forms [130]. Another issue with FDM is that pharmaceutical polymers have been developed for processability in traditional processes but not in FDM. Filaments require certain mechanical properties to make them feedable and printable, and several pharmaceutical polymers pose challenges in this regard [131,132]. The authors believe that these risks and downsides are easily compensated for by the novel opportunities of FDM. If the extruded filament fulfills all critical attributes, FDM can be considered the easiest to use and most robust 3D printing technology.

#### 4.1.4. Electrospinning of ASDs

Descriptions of the effects of electrostatic attraction on liquids date back to 1600 [133]. However, the concept of electrospinning took some time, with the first patents on electrospinning and suitable devices filed in the late 19th and early 20th century by Cooley [134], Morton [135], and Formhals [136]. Since then, the process has become increasingly refined, and research and industrial scale-up efforts have resulted in over 45,000 publications as of the end of 2024 and created many different usage forms. Overall, the amount of articles on electrospinning has shown exponential growth since 2001 [137]. Whereas the basic setup of an electrospinner can be broken down into four components—high-voltage power supply, conductive collector, syringe pump, and spinneret—the process parameters are numerous and complex. Regarding the material to be electrospun, the process can be divided into the following three main types: melt, solution, and emulsion electrospinning. All three can be used to prepare fibers applicable as a drug delivery system, with the second being the preferred method because of its easy use.

**Figure 6:**
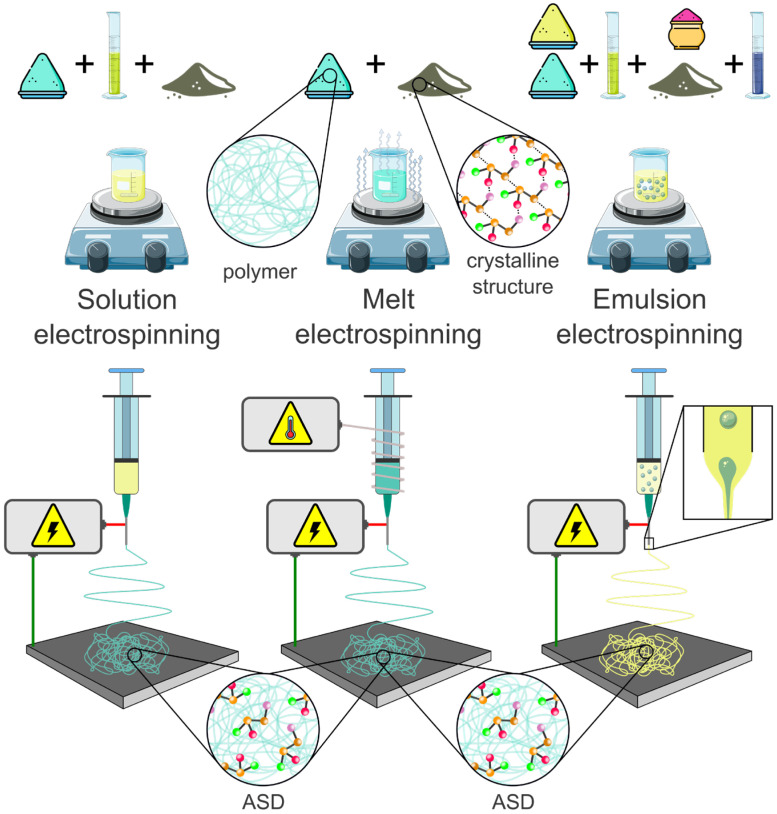
Different methods using electrospinning for ASD preparation.

Almost all polymers or substances (hydrophilic or hydrophobic) can be electrospun by one or all methods. The fibrous meshes are commonly classified as solid dispersions, and because of the high level of amorphousness resulting from the preparation process, they are often further categorized as ASDs [138]. Various excellent reviews discuss the physical principles [139,140], different setups [137,141], and release profiles of various ASD types [138]. Hence, this section highlights the advantages and drawbacks of melt (MES), solution (SES), and emulsion electrospinning (EES) (Figure 6). Furthermore, we evaluate and discuss the most recent developments in terms of the amorphousness of an encapsulated drug and polymer and the release profile.

a. Solution electrospinning (SES)

One of the first and easiest ways to prepare micro- or nanofibers via electrospinning is to dissolve the chosen polymer in an organic or aqueous solvent and electrospin the solution, adjusting direct process parameters like applied voltage, solution flow rate, and the distance between the spinneret and collector. Considering the diverse solution and environmental parameters already described in detail elsewhere, achieving smooth fibers without bead defects might be challenging. However, if the correct settings are found, fiber preparation can be performed conveniently and reproducibly. Based on this, diverse attempts have been made to modify the preparation method to meet the requirements of different drugs, their medical indication, and the requirements of industrial scale-up. In general, SES necessitates the drug and polymer matrix to be dissolved in the same solvent. A high release rate can be expected in most cases because of the high surface-to-volume ratio of a fibrous mesh (especially in nanofibers).

Regarding their indication, complete depletion of a drug at the beginning of the treatment might not always be the right choice. Thus, alternative setups of the spinneret (e.g., coaxial, triaxial, and side-by-side electrospinning) were invented, broadening the possibilities. Another attempt was made by introducing previously prepared nanocarriers into the dissolved polymer solution, overcoming the restrictions imposed by the prerequisite of similar solubilities of the drug and polymer. Altogether, the variety of possible combinations can cover many indications. Regarding industrial scale-up, multi-needle spinnerets and needleless setups made upscaling and continuous manufacturing possible. Despite numerous advantages, some gaps could only be covered by other modifications (Table 1).

b. Melt electrospinning (MES)

Compared with SES, MES is a relatively neglected topic. In MES, no organic solvent is necessary, earning it the term ‘green option’ by some researchers. The polymer is liquified by an adequate temperature input, enabling the proper flow through a spinneret. According to a web search, using www.webofscience.com, the umbrella term ‘electrospinning’ returned 45,536 publications between 2001 and 2024, whereas ‘melt electrospinning’ was only mentioned 390 times. Less than 10% of both the electrospinning and MES publications contain the term ‘drug delivery’, with approximately twice as many of the latter being review articles.

Unfortunately, only a handful of research articles directly compare SES and MES using the same polymers and drugs, complicating a direct comparison of both regarding drug delivery. Nagy et al. and Balogh et al. incorporated carvedilol, a BCS class II drug, into Eudragit^®^ E and PVP/VA 64, respectively, via MES and SES. Polymers and carvedilol demonstrated amorphous characteristics, leading to a high release rate in both studies [142,143]. Lian et al. loaded increasing concentrations of curcumin, a BCS IV drug, into poly-ε-caprolactone (PCL) fibers. Whereas curcumin turned out to be amorphous using MES and crystalline using SES, it was the other way around for the polymer matrix. PCL’s crystallinity was higher in MES fibers than in SES fibers, resulting in a higher release rate by the latter [144]. Depending on the drug and polymer, similar or diverse degrees of crystallinity of the polymer and drug can be achieved, broadening the outcomes of the release profiles. Further investigations are necessary to enable predictions and more elaborate decision making.

c. Emulsion electrospinning (EES)

One of the first articles on EES was published in 2006 by Xu et al., who electrospun a W/O emulsion of poly(ethylene oxide) in a poly(ethylene glycol)-poly(L-lactic acid) (PEG-PLA) diblock copolymer [145]. They could prepare core–sheath nanofibers comparable to coaxial electrospinning using a single-needle spinneret. Since then, a simpler equipment setup requiring more complex material preparation has been utilized to achieve core–sheath nanofibers, shifting the workload from process to solution parameters and bringing other advantages and disadvantages [146] (Table 1).

Depending on the medical indication, various release profiles were adapted, going from one extreme to another. By electrospinning an O/W nanoemulsion, Kamali et al. could encapsulate curcumin (BCS IV) in PVP K90 (hydrophilic polymer) fibers. Ty achieved an instantaneous release within the first 30 s, making it applicable as a fast-dissolving oral film [147]. In contrast, Shibata et al. were able to control the release of probucol (BCS II) from PVA nanofibers. They evaluated the effects of the PVA grade, used oil phase, and surfactant concentration on the release pattern. Compared with the simple SES method, which resulted in a burst release of probucol, they achieved a sustained release by fine-tuning EES parameters [148].

d. Current trends in electrospinning

Electrospun fibers can be used in many ways, but in drug delivery, the focus in mainly on the drug release profiles. Current trends still follow the goal of adjusting the release to the intended application; however, recent advances also focus on the subsequent processes after the release (e.g., degradation, bioavailability). Yu et al. were the first to prepare electrospun PVP K60 fibers capable of the self-assembly of phosphatidylcholine (PC) liposomes when coming in contact with water. The concentration of PC had a direct effect on the liposome size [149]. Friedl et al. improved this idea and fabricated self-emulsifying drug delivery system (SEDDS)-loaded fibers (Section 5.3.2 for SEDDS). They combined Kolliphor EL, Capmul MCM, Captex 355, and Transcutol with Resomer 503H and PVP and electrospun the solution. The release of the model compound curcumin could be controlled by varying the concentration of the SEDDS in the fibers [150]. Ge et al. compared uniaxial and coaxial electrospinning and prepared SEDDS-loaded fibers with paclitaxel (BCS IV). Based on their results, they suggested that the core–shell fibers showed a better performance [151].

Another underrepresented but trending topic are Janus fibers. Janus fibers are two-compartment systems in which both compartments are in direct contact with the environment. Compared with their counterparts—core–shell fibers—as two-compartment systems, Janus fibers create other exciting options in drug delivery. As both compartments are in direct contact with their environment, other release profiles and tunable systems can be designed. Yu et al. prepared side-by-side fibers with PVP K60 (+ketoprofen) on one side and ethyl cellulose (EC) (+ketoprofen) on the other side. Higher release rates could be achieved by adding and increasing the PVP K10 concentration on the EC side [152]. In two consecutive publications, Wang et al. electrospun side-by-side zein–PVP fibers with a two-stage controlled release and, later, even prepared chimeric tri-layer side-by-side fibers [153,154].

### 4.2. Crystalline Dispersions

Crystalline dispersions enhance the bioavailability and therapeutic efficacy of poorly water-soluble drugs by improving their dissolution. Compared with ASDs, this effect is not attributed to the solid state of a drug but results from embedding a crystalline drug in a hydrophilic carrier matrix. This matrix increases the wettability of the drug so that the dissolution is accelerated [155]. The speed of dissolution can be further amplified by decreasing the size of the drug particles in the carrier matrix. The underlying mechanisms of increased bioavailability for smaller particles have already been described for the nanoparticle formulations above (Section 3.1) and are mainly attributed to the increased surface area, which improves the dissolution kinetics. In this section, the focus is on the superimposed influence of the hydrophilic carrier matrix and the particle size reduction. The handling of small drug particles in the nanometer range requires special measures to achieve their full therapeutic potential and prevent agglomeration. In this context, the carrier matrix captures single drug particles in place and keeps them away from each other so that the advantageous effect of small drug particles is preserved. Furthermore, crystalline dispersions are thermodynamically stable, which offers a long shelf-life. Several processing techniques have been employed to prepare pharmaceutical crystalline dispersions, including spray drying [156], extrusion [157], and melt milling [30], and are partially discussed above (Section 3.1.3).

Co-crystal formulations are a special type of crystalline dispersions. Co-crystallization techniques utilize co-formers to modify the crystalline structure of drugs [158]. The formation of crystalline complexes between a drug molecule and a co-former modulates its physicochemical properties, including solubility, dissolution rate, and stability. The selection of an appropriate co-former and an understanding of molecular interactions are crucial for designing co-crystals with the desired properties and therapeutic effects. The field of co-crystallization continues to evolve rapidly, driven by advances in formulation science, computational modeling, and materials characterization. Current trends include the exploration of novel co-formers, the development of rational design approaches, and the integration of co-crystals into personalized medicine strategies. Future research directions may involve elucidating the mechanistic insights underlying co-crystal formation, optimizing co-crystal synthesis methodologies, and advancing clinical translation to realize the full therapeutic potential of co-crystallization in diverse disease settings. Processing technologies are intended to facilitate intimate mixing and close contact between the drug and co-former molecules, which are critical for the formation of co-crystal bonds [101].

### 4.3. Mesoporous Systems and Aerogels

Mesoporous carrier systems are an emerging alternative approach to polymer-based systems to keep poorly soluble substances in an amorphous state, which favors improved dissolution and bioavailability. These systems arose in the early 1990s. In such mesoporous systems, which may be made of inorganic or organic materials, a high specific surface area and so-called mesopores in the range of 2 to 50 nm prevail. They provide massive surface interaction potentials and spatial confinement within the pores, which thermodynamically hinder the crystallization of embedded drugs. According to the mentioned structural properties, mesoporous carriers possess high porosity and low density, which is why they are frequently also referred to as aerogels.

In pharmaceutical approaches, mesoporous systems made of organic materials, such as alginate [159], starch [160], or cellulose [161], as well as inorganic materials, are applied, under which silica systems are those most frequently used [162,163,164]. Such amorphous silica is harmless to humans and generally recognized as safe for oral delivery. Hybrid materials combining silica and alginate further broaden the material properties and application by integrating the mechanical strength of silica with the biodegradability of alginate [165]. The predominance of silica as a mesoporous drug carrier system is based on its versatility in the geometric and chemical tunability of pore dimensions and networks, as well as its surface chemistry, due to the presence of silanoles as starting groups [164,166]. Commercial mesoporous systems, especially different types of Syloid^®^ and Neusilin^®^, are available [167].

The synthesis processes of aerogels are not discussed in detail and can be found elsewhere [162,163,167,168]. In brief, sol-gel synthesis, in which aerogels are finally yielded by supercritical CO_2_ extraction, and surfactant-templated self-assembly, in which the structures of micelles or liquid–crystalline structures act as spacers for the pores and are chemically or thermally removed from the silica scaffold after synthesis, can mainly be differentiated. The surface functionalization of mesoporous silica is applied to tune the interaction between drug molecules and the carrier with the aim of enhancing the loading capacity and controlling the drug release rates [164,166]. The stabilization of the amorphous state of drugs in mesoporous silica is frequently associated with a reduced molecular mobility due to special confinement, a lower propensity for nucleation, a reduction in Gibbs free energy due to contact with the high surface energy of silica, and specific interactions of the H-bonding with its superficial silanol groups [167].

For embedding poorly soluble drugs into mesoporous systems, it is most important that extensive surface contact between the drug (molecules) and the carrier surface is facilitated. This includes bringing the mostly crystalline drug into an amorphous state, which can generally be achieved via energy input in solvent-free approaches or via dissolution of the crystals in suitable fluids in solvent-based approaches. Overall, a multitude of methods for drug loading into mesoporous carriers have been studied, each influencing the final drug-loading capacity, release properties, and stability of the drug [162,163,164,167,169,170]. The drug can be introduced into the carrier system at different stages during or after its synthesis. During carrier synthesis, drugs can be introduced into the precursor solution or gel or during the final synthesis step of supercritical drying with CO_2_ [159,171,172,173], in the case that this drying step occurs during carrier synthesis. However, drug loading during synthesis may be critical based on the chemical nature of the drug and whether it can withstand the synthesis conditions without side reactions with the precursors or degradation under the necessary conditions.

In order to prevent chemical reactions during carrier synthesis, post-synthesis loading of pre-formed mesoporous carrier materials can be realized. This may also be the industrially more realistic and economic approach, as the synthesis of the carrier (excipient production) and the loading of the drug (dosage-form manufacturing) can be divided, as is common. Depending on the susceptibility of the drug, solvent-based methods can be applied for heat-sensitive substances—although solvent-free energy input-based methods are commonly more efficient—and they may yield higher drug loadings, as well as being more scalable. Among solvent-based methods, the supercritical CO_2_ impregnation with the drug resembles the final drying step in aerogel synthesis. Applying this method, the achieved drug loading in the carrier is often low and, crucially, depends on the solubility of the drug in supercritical CO_2_ [174,175,176]. Other solvents that are liquid under ambient conditions, such as dichloromethane, may be applied in drug loading [162,163,177,178]; however, residual solvents remain a challenge for product development and quality control. The application of a drug solution in carriers can be conducted in a high excess of solvent, relying mainly on molecular adsorption onto the carrier, or without an excess of solvent (i.e., incipient wetness method) with highly concentrated drug solutions, which is meant to dispense higher doses of the applied drug directly into pores. The latter can also be combined with efficient drying methods, such as rotavapor, fluidized bed [179], or spray drying [180].

Solvent-free loading combines the challenge of the amorphization of the drug by controlled energy input with the potential of efficient processing and scalability for industrial production. The energy input can be achieved by direct or indirect introduction of heat. Most simple melt-based approaches consist of the powder blending of the drug and carrier, with subsequent elevation of the temperature in containers [177,180,181,182]. Thermal loading methods have been further developed toward applicability on larger scales using a fluidized bed setup to melt and disperse the drug particles to make contact and be absorbed into carrier particles [183]. Thermal energy can also be introduced by radiation, such as microwaves to amorphize drugs in carrier blends [184]. Some of the previously introduced solvent-based and non-mechanical physical methods have been compared regarding their loading and release kinetics, showing distinct differences depending on the loading method [185].

Further advances in continuous loading methods have been made by studying the processability via hot-melt extrusion of dry powder blends [186], which combines thermal and mechanical energy inputs. It was proven that, depending on the processability of the carrier, hot-melt extrusion can be applied. However, the classical approach to mechanically introducing energy to support the amorphization of drugs in blends with mesoporous carriers is co-milling [187,188]. Herein, drug and carrier powder are blended and, in most cases, stressed by means of mills, applying a grinding media. Most of the energy is dissipated to the particles by means of plastic deformation and heat, which can, in turn, locally facilitate the amorphization and imbibition into the carrier particles. However, comparing the solvent deposition and co-milling showed that, depending on the carrier used, the loading method has an effect on the loading capacity, as well as the dissolution rate, with lower values for the co-milling approach [189]. These effects were traced back to the reduction in pore volume of the carrier due to the mechanical stresses from co-milling. Other studies showed that the physical state of the loaded drug in the pores depended on the loading method, as well as the chemical nature of the drug [179,190].

The loading capacity is generally governed by the specific surface area, mesopore volume, pore size (distribution), pore structure (e.g., ordered or unordered and interconnectivity), and chemical surface properties [164,167,191]. The resultant release rates from mesoporous systems may be up to ten times quicker than for pure drug substances [175,192]. With chemical surface modifications, the release may also be sustained depending on the interactions with the drug [164,176,193]. Additionally, higher amorphous drug loading may also result in a slower release of the drug [167]. The storage stability may be influenced by the abovementioned parameters, as well as the ratio between the critical nucleus size of the drug to be crystallized and the mesopore diameter [194].

Subsequent to the drug loading in the mesoporous carriers, there is the need for further formulation and processing into the final dosage form such as tablets. During tableting, mechanical stresses may again compromise the pore volume of the mesoporous carriers. Nonetheless, the literature reports that improved dissolution kinetics by amorphous loading into mesoporous systems can also be retained after tableting [179,195]. However, mesoporous systems may negatively alter the mechanical properties of tablets [196], producing softer tablets due to the loss in compatibility. Further application strategies for orally delivered mesoporous systems include the formulation of gastric-floating dosage forms [197], loading capacity enhancement of individualized orodispersible films [198], and the enhancement of the loading capacity of amorphous poorly soluble drugs in the 3D printing of pastes [199].

Finally, it should be mentioned that mesoporous silica has also been studied in nanodrug delivery devices in parenteral applications to overcome biological barriers [200] and in cancer treatment [201], as well as against bacteria and their resistances against antibiotics [202].

## 5. Lipid-Based Formulations

Lipid-based formulations are often used to formulate ‘grease-ball’ compounds with high logP values. They can be associated with physical modification methods, as they utilize non-covalent encapsulation mechanisms. However, the complex interactions between drugs and lipids suggest that these formulations represent a hybrid approach that bridges the gap between purely physical and chemical modification strategies.

Various lipids can be used in these formulations, such as phospholipids, cholesterol, and long- or medium-chain mono-, di-, or triglycerides, as the sole substance or in combination with surfactants. Depending on the utilized additives, some formulations may also have self-emulsifying properties [203,204]. A classification system was introduced in 2000 to enable better evaluation of the critical performance characteristics of lipids as excipients. The LFCS (lipid formulation classification system) divides lipid formulations into Types I to IV. While Type-I formulations consist only of oils, Type-II and -III systems are self-emulsifying drug delivery systems with water-insoluble and water-soluble surfactants, respectively. Formulations with hydrophilic surfactants and co-solvents are classified as LFCS Type IV [205,206].

The first positive effects of lipids on the bioavailability of poorly water-soluble drugs were reported in 1961, when it was shown that the serum concentration of griseofulvin almost doubled after a high-fat breakfast [207]. Later studies showed that lipids could positively influence the intestinal permeability of drugs, whereby a formulation-dependent increase in passive paracellular or transcellular permeation was detected. Paracellular transport occurs when the drug passes through the intercellular gaps between the epithelial cells, which are regulated by the tight junctions. During transcellular permeation, a drug is transported into the cell by membrane vehicles [204]. Furthermore, lymphatic transport by nanoparticular lipid formulations that bypass the first-pass effect in the liver has been reported [208].

Recent studies have reported that various factors can influence the permeability of lipid-based formulations. In addition to the size and shape of lipid particles, the surface charge, surface modification, and type of lipid have a significant impact. It has been shown that oral bioavailability is improved with digestible lipids, such as triglycerides, cholesterol, and phospholipids. The body can assimilate the digested products after hydrolysis. Indigestible lipids (e.g., mineral, essential, and flavor oils) are not hydrolyzed, which limits their positive influence on the bioavailability of drugs. However, these lipids can improve the stability of a nanoparticular formulation. Therefore, combining digestible and indigestible lipids may be advantageous [208]. Differences were also found in relation to the chain length and degree of unsaturation. It was shown that medium-chain triglycerides improve absorption better than long-chain triglycerides, which degrade more slowly [206].

In addition, the enhanced solubilization of lipophilic drugs could increase exposure and reduce food effects, which are often observed after oral administration of poorly water-soluble drugs [209]. Another advantage of lipid-based formulations is that lipids are physiological and, therefore, have a high tolerance in the human body. No toxic co-solvents or pH adjustments are required to solubilize lipophilic drugs in such carrier systems [204]. Because the drug is usually dissolved in lipid-based formulations, they are mainly suitable for low-dose components [209].

**Figure 7:**
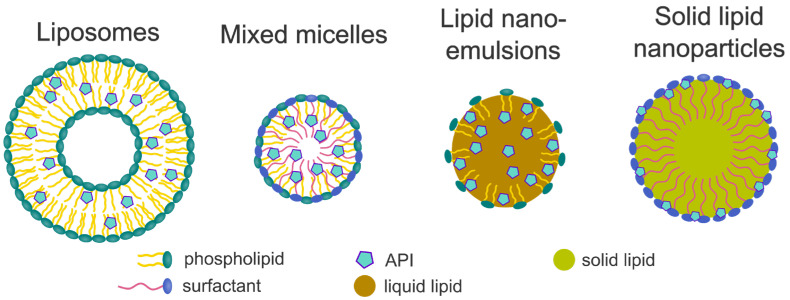
Exemplary illustration of drug-loaded lipid-based formulations.

In general, lipid-based drug delivery systems are divided into liposomes, micelles, lipid dispersions, such as emulsions and suspensions, and self-(micro/nano)emulsifying systems [204,210] (Figure 7). Nanostructured lipid carriers, which consist of a mixture of liquid and solid lipids, play a rather subordinate role and are not discussed further. In this last section, the latest research progress regarding these different types of formulations is discussed. Although liposomes and micelles are rarely used in the formulation of solid oral dosage forms, they have been included in this review as they represent important lipid-based approaches for poorly water-soluble drugs.

### 5.1. Liposomes

The success story of liposomes goes back to the mid-1960s, when Bangham et al. first described ‘liquid crystals of lecithin’ that exhibited similar diffusion properties for ions as biological membranes [211]. Being then first used as artificial models for biological membranes, these newly discovered vesicles were soon identified as promising carriers for enzymes and drugs [212,213,214,215]. Their unique structure enables the encapsulation of both hydrophobic and hydrophilic cargoes. After another decade of extensive research in this field, the development of liposomal formulations finally resulted in the first in vivo studies in humans with amphotericin B and doxorubicin in the late 1980s [216,217] and in the clinical application of AmBisome^®^ and Doxil^®^ since the 1990s [218,219]. Their ability to carry a wide range of compounds, including drugs, biologicals, and imaging agents, makes them valuable in both drug delivery and therapeutic applications.

#### 5.1.1. Composition of Liposomes

One of the key components of liposomes are phospholipids. Phospholipids are based on a glycerol backbone with fatty acids esterified at the C1 and C2 positions, as well as a phosphate group at the C3 position. This structure gives phospholipids amphiphilic properties, making them valuable natural solubilizers, wetting agents, and excipients for the formation of (mixed) micelles and liposomes [220]. Phospholipids consist of a hydrophobic tail formed by fatty acids and a hydrophilic head composed of the phosphate group, which is typically further esterified with an additional alcohol, such as choline, ethanolamine, or inositol. This structure allows phospholipids to form vesicles that closely resemble the structure of cell membranes when dispersed in water. In the case of liposomes, phospholipids assemble to form a single or several concentric bilayer structures, with the hydrophobic tails forming a lipid bilayer and the hydrophilic heads facing both the aqueous core and the surrounding aqueous medium. Based on this structure, liposomes are capable of including both hydrophilic cargo molecules in the aqueous core and hydrophobic cargo molecules in the lipophilic bilayer. As liposomal bilayers are fluidic systems, incorporated drugs may leak or be released over time, which can result in unwanted stability issues but also offers the opportunity to modify the drug release [221]. Additional therapeutic benefits, such as a prolonged half-life in the blood stream, improved stability in the gastrointestinal tract, or a targeted delivery of the active drug to, for example, specific tumor tissues, may be achieved by modifying the liposome surface [221,222]. Thus, apart from improving the solubility of poorly water-soluble compounds, drug encapsulation in liposomes is also used to improve drug stability, alter drug pharmacokinetics, or to enable drug delivery to target cells or tissues, making liposomes a very versatile drug delivery technology [223].

#### 5.1.2. Preparation of Liposomal Formulations Containing Poorly Water-Soluble Drugs

Over the years, several different techniques for obtaining liposomal formulations have been reported. As this review aims to provide formulation approaches for improved bioavailability of poorly water-soluble compounds, this section only addresses liposome preparation methods that are primarily capable of incorporating lipophilic drugs into vesicular systems. The most used approach for this purpose is the film hydration technique. In this process, lipids and the lipophilic drug are dissolved in an organic solvent. The solvent is then removed, e.g., by rotary evaporation. Afterwards, the resulting film is hydrated by adding aqueous (buffer) media, which mostly results in the formation of multilamellar vesicles that are then subjected to a homogenization step for reductions in both the particle size and particle size distribution. Another approach, referred to as reverse-phase evaporation, is to directly mix the lipophilic component solution in organic solvents with water to form an emulsion and then to evaporate the organic solvent. With microfluidic methods, liposomes can also be prepared without organic solvent use. In this technique, the lipid phase is directly mixed with the aqueous phase, allowing for precise process control regarding the particle size, vesicle charge, and surface modification [221]. An alternative methodology for conducting the homogenization process in vial is dual centrifugation. This innovative technique, first described by Massing et al., employs a centrifugation mechanism that includes an additional rotational movement of the sample along its vertical axis. This approach generates substantial shear forces, thereby enhancing the efficiency of the homogenization procedure for liposomal formulations [25,224].

A significant limitation of these methods is the relatively low entrapment efficacy, meaning that the drug cargo is not completely incorporated into vesicles [225]. For hydrophilic and ionizable drugs, various techniques have been proposed to actively load the cargo into liposomes. As the drug is loaded into liposomes by a specific trigger after the vesicles are formed, these approaches are also referred to as active loading. Most of these methods leverage the non-ionized state of the drug to facilitate its passage through the lipid bilayer into the aqueous core, where it is directly ionized and trapped. Some principles for actively entrapping hydrophilic drugs inside the aqueous liposome core include the utilization of pH gradients, (calcium)-citrate-based methods, and ammonium sulfate gradients, among others [225]. Considering the loading of liposomes with lipophilic compounds, there are few cases where poorly water-soluble drugs have been remotely loaded into liposomes. A prominent example of a poorly water-soluble drug in a liposomal formulation is paclitaxel, which has been a subject of interest for formulation scientists for decades. While conventional preparation strategies such as thin-film dispersion or extrusion are accompanied with a low entrapment efficacy of approx. 50%, recent advances allowed for significantly increasing the amount of paclitaxel entrapped in liposomal formulations. Recently, two methods for the active loading of liposomes with paclitaxel based on a calcium acetate gradient were reported. Since the mother compound, paclitaxel, is a non-ionizable drug, in the first instance, weak acid derivatives with succinic or phenylboronic acid were synthesized, which were then loaded into aqueous cores of liposomes. Using this approach allowed for the achievement of a notably high encapsulation efficiency of more than 95% [226,227]. In another study, Yu et al. utilized a paclitaxel–doxorubicin prodrug, featuring an anthracene structure capable of interacting with copper ions. By implementing a copper ion gradient, they successfully loaded the paclitaxel–doxorubicin prodrug into liposomes, facilitating a combination therapy involving these two poorly water-soluble anti-cancer agents [228].

### 5.2. Mixed Micelles

As early as 1909, Moore et al. observed that the addition of lecithin to a solution of bile salts (BS) resulted in the formation of a transparent solution rather than an emulsion or suspension [229]. Furthermore, this solution demonstrated enhanced lipid solubilization properties compared with individual BS or lecithin solutions. In addition, further studies proved that the addition of equimolar amounts of phospholipids to BS solutions resulted in the mitigation of the hemolytic and cytotoxic effects of BS solutions [230,231,232]. The resulting species that derived from mixing BS and phospholipids components, mixed micelles (MMs) exhibit a spherical structure with a hydrophilic surface and hydrophobic core that enable the entrapment of lipophilic compounds (Figure 7). Physiologically, MMs play a key role as natural solubilizers in fat digestion and absorption, but with their amphiphilic structure they can also act as carriers for lipophilic xenobiotics. Based on these structural requisites, MMs also became the focus of formulation scientists as a potential solubilization technology for use in pharmaceutical development. In the early 1970s, initial attempts were made to use MMs in pharmaceutical formulation development for lipophilic and poorly water-soluble drugs. This resulted in the approval of a diazepam MM formulation (Valium^®^ MM) by Hoffmann-La Roche in 1976 [233]. Since then, MM formulations have been investigated for several poorly soluble drugs across various therapeutic areas, including analgesics, sedatives, glucocorticoids, steroid hormones, immunosuppressants, and cytostatic drugs [234,235,236,237]. However, only a few MM formulations, such as Cernevit^®^ (a vitamin blend) or Konakion^®^ MM (a vitamin K1 formulation), have gained marketing authorization and, to date, only Konakion^®^ MM is still in use as a liquid MM formulation that can either be given via the parenteral or the oral route.

The combination of both an excellent toxicity profile of necessary excipients and the versatile use of a single formulation for various routes of administration make MMs a very promising formulation approach for the development of pediatric formulations [238]. Many enabling technologies that are frequently used in adult formulation development make use of excipients, such as synthetic polymers or surfactants, for which evident toxicity data in children are still lacking. While MM formulations containing phospholipids and BS as costly excipients are less attractive than other more cost-effective approaches for adult formulations, MMs may close an important gap in pediatric formulation development. Excipient safety is one key aspect that needs to be specifically addressed when developing novel and state-of-the-art pediatric formulations [239]. The excipients used in MM formulations, phospholipids and BS, are endogenous substances and, thus, come with little to no safety concerns.

#### 5.2.1. Liquid MM Formulations

The solubilizing properties of MMs are very drug-specific and strongly depend on formulation parameters such as the applied phospholipid/BS ratio or the MM concentration. Thus, when developing a novel MM formulation, an initial formulation screening (i.e., determining a formulation composition that allows for maximum drug solubilization) is mandatory. The most widely used method for preparing MM formulations in this screening stage is thin-film dispersion. In this process, the individual components (i.e., phospholipids, BSs, and the drug) are dissolved in organic solvents and mixed at predetermined ratios. In a following step, the solvent is then removed by means of rotary evaporation, and the resulting phospholipid/BS/drug layer is redispersed in a suitable solvent, such as water or a buffer solution, in an inert atmosphere to finally obtain a drug-loaded liquid MM formulation. To obtain MM formulations in a solvent-free process, which should be favored for large-scale production, they can also be prepared in a different manner. At low concentrations, the physiological benefit of MMs as mild solubilizing agents impedes drug loading in the formulations, since MMs are unable to overcome the lattice energy of crystalline drugs to enable drug dissolution. To increase the solubilizing properties of the MM components as part of the production process, there are mainly two ways to prepare MM formulations without organic solvents, as follows: dissolving the drug in highly concentrated BS solutions followed by the addition of phospholipids or the use of highly concentrated MM solutions that are terminally diluted to the target MM concentration after previous drug loading [238].

#### 5.2.2. Solid MM Formulations

All former and presently approved MM formulations are primarily liquids for parenteral use, but due to stability concerns, processes to transform liquid MM formulations to solid, with lyophilization being the most prominent, have been studied. With Cernevit^®^, the marketing authorization level was reached [240]. With the aim of obtaining oral formulations, the incorporation of MMs in more convenient solid oral dosage forms, such as pellets, mucoadhesive buccal tablets, or fast-dissolving oral films, containing MM precursors and forming MMs after redispersion upon contact with saliva or gastrointestinal fluids was also addressed on an investigational basis but not intended to undergo the approval process [222,241,242,243].

### 5.3. Lipid Nanoemulsions

#### 5.3.1. Nanoemulsions

In the current context, lipid nanoemulsions are commonly known as dispersions of drug-loaded lipids in an aqueous phase. Caused by their structure, they enable a higher solubilization capacity than micellar systems [244], and the drugs can be localized in the liquid core, droplet interface, and aqueous phase [245,246]. Lipid nanoemulsions have been used in a pharmaceutical context for over five decades and were introduced for parenteral application in patients who are unable to take food orally [247]. For parenteral use, the droplet sizes are strictly controlled to avoid embolisms and, thus, are often in the range between 200 and 400 nm [248]. These formulations are not restricted to only one application route but could be used for different delivery modes, such as topical, ocular, intranasal, or peroral [244,249].

The preparation of lipid emulsions requires successfully overcoming the interfacial tension between the two immiscible liquids. Therefore, an energy input is required, which is often based on high-energy methods. Sonication devices are mostly used in a research environment. The generated vibrations lead to pressure fluctuations in the fluid and cause bubble cavitation, resulting in high shearing energy. Rotor-stator, as well as high-pressure, devices are common in large-scale manufacturing [250]. Another possibility to introduce high shear forces into a formulation is the use of grinding media. This can be conducted, on the one hand, by applying a stirred media mill [251] and, on the other hand, by dual centrifugation in a small-scale approach of approx. 1 mL [25,252]. Although all of these technologies enable the manufacturing of emulsions as soon as the interfacial tension is overcome, these material systems are highly instable, which manifests in various destabilizing mechanisms such as flocculation, Ostwald ripening, and coalescence [250]. Stabilization of a formulation is possible by adding stabilizing agents such as surfactants to the aqueous phase to decrease the interfacial tension and prevent destabilization. Nanoemulsions, in particular, are known to be stable against sedimentation and creaming due so their small droplet sizes, and Ostwald ripening appears to be one of the main causes of a breakdown in the stability of O/W emulsions [253].

Drug loading in lipid emulsions can be performed actively prior to the emulsification process or passively by an incubation step after the emulsion’s preparation. Passive drug loading is advantageous for small setups such as screening approaches, while carrier overloading, possibly resulting in drug recrystallization during storage, was not observed [254]. During active loading, the drug is directly dissolved in the liquid lipid prior to emulsification. As a result of both manufacturing approaches, the drug is dissolved in the dispersed lipid phase of the emulsion in order to enhance the bioavailability of the poorly water-soluble substances. Additionally, it was shown that the encapsulation of the drug molecules in the lipid droplets could prevent oxidation and hydrolysis processes [244,255].

Focusing on the peroral delivery of poorly water-soluble drugs, as this application route is most preferred by patients [256], the positive effects of lipid-based nanoemulsions have been recorded. An increase in antimalarial activity was shown for primaquine when loaded in lipid nanoemulsions, resulting in 25% lower doses [257]. Furthermore, an improved anti-inflammatory effect was identified for a curcumin-loaded lipid nanoemulsion [258], and improved oral bioavailability was reported for drugs such as paclitaxel (dispersed in pine nut oil) [259], breviscapine (dispersed in ethyl oleate) [260], and pterostilbene (dispersed in isopropyl myristate) [255]. So far, most of the lipid-containing systems under investigation are liquid formulations. But studies showed that the lipid nanoemulsions can also be further processed to semi-solid or solid dosage forms. Yu and Huang formulated organogel-based nanoemulsions loaded with curcumin [261], and in other studies, the emulsions were further processed to dry powders by spray drying. These showed that improved oral bioavailability was achieved when the drugs itraconazole [262] or 5-PDTT (5-phenyl-1,2-dithiole-3-thione) [263] were formulated in lipid emulsions and embedded in matrixes during spray drying. In addition, studies showed that the nanoparticular properties of lipid emulsions could be preserved after spray drying, independently of the used oil, when embedded in a matrix containing lactose and the surfactant SDS (sodium dodecyl sulfate) [264]. Furthermore, the latest studies show that lipid nanoemulsions can also be embedded in an orodispersible film matrix. Although the nanoparticular properties of the lipid droplets could not be completely preserved when using HPMC as a film-forming polymer, dissolution studies showed an increased dissolution rate for the embedded fenofibrate [67].

#### 5.3.2. Self-(Micro/Nano)emulsifying Formulations

Unlike nanoemulsions, self-(micro/nano)emulsifying drug delivery systems (nomenclature used is SEDDS, SMEDDS, or SNEDDS) form O/W emulsions when they come into contact with an aqueous phase. The first self-emulsifying drug delivery systems were formulated in the 1970s and 1980s [265,266] using, for example, medium-chain triglycerides and non-ionic surfactants [266] encapsuled in gelatin capsules to enable oral administration [265].

Classic SEDDS contain liquid oils in which the drug is dissolved. Modified or hydrolyzed vegetable oils have been used to improve the success of systems by enabling higher drug loadings. With a share of 30 to 60%, surfactants play an important role in a formulation. Mostly non-ionic surfactants with a high hydrophilic–lipophilic balance (HLB) such as Polysorbate 80 are used [267]. Due to their high share in formulations, emulsifiers from natural sources are expected to be safer, and recent studies have successfully used, for example, phospholipids such as lecithin, which has been classified as GRAS (Generally Recognized As Safe) by the FDA [268]. To improve the solubility of the drug or the surfactant in the lipid, co-solvents such as ethanol or propylene glycol can be added to formulations [267]. During a formulation’s preparation, the dissolution of the drug in the lipid formulation can easily be achieved with low-viscous lipids by, for example, simple stirring processes. However, it is more challenging when lipids with high viscosities or semi-solid formulations are to be formulated. In some cases, this could be solved by higher process temperatures, but this requires the usage of no thermosensitive drugs or excipients. A preparation technique that proved to be particularly suitable for highly viscous systems was introduced by Gruene and Bunjes in 2024. They used dual centrifugation to prepare formulations with five different poorly water-soluble drugs, where the applied centrifugal forces intensify the mixing process [269].

The majority of the SEDDS formulations are applied perorally in patients [270], and the lipid-based, drug-containing formulation is encapsuled in hard or soft capsules [271]. Recent studies show that SEDDS can also be encapsuled in more innovative dosage forms. For example, self-microemulsifying mouth-dissolving films were introduced by Xiao et al. in 2011 [272] and later used in the formulation of the poorly water-soluble drug indomethacin [273]. In a 2019 study, Talekar et al. prepared self-nanoemulsifying orodispersible films (SNEODF) using the BCS class III drug captopril [274]. They formed a stable W/O/W nanoemulsion from the oil phase and an external phase containing the film-forming polymer HPMC, a plasticizer, and water and casted a film from this mixture. Solid self-emulsifying drug delivery systems (S-SEDDS) were introduced by Abdalla and Maeder in 2007 [275], which were further developed in the 2020s by 3D printing oral tablets. Vithani et al. published a proof of concept for the preparation of S-SEDDS in four different shapes loaded with fenofibrate and cinnarizine by printing the lipophilic formulation at 65 °C using a printer-mounted syringe [276]. In addition to printing the molten lipid formulation [277], it was shown that emulsion gels can also be successfully formulated from self-emulsifying formulations and 3D printed [278]. However, in addition to peroral application, other routes of application for SEDDS have also been investigated, such as intranasal application, to allow for better targeting of the brain, which is not discussed in this review but can be referred to in [270,279].

### 5.4. Solid-Lipid-Based Formulations

Compared with lipid nanoemulsions, lipid nanosuspensions are prepared from a lipid with a crystallin structure at room temperature. The first publications regarding solid lipid nanoparticles go back to the year 1992, where Lucks et al. introduced lipid particles as an alternative parenteral drug delivery system [280]. Lipid nanoparticles are mainly prepared by techniques such as solvent injection, ultrasound technique, microemulsion technique, or high-pressure homogenization [281,282]. Most technologies require a melting of the lipids above their melting point prior to processing to enable emulsification with the aqueous phase. Crystalline particles are formed from the lipid droplets when cooled afterwards. In terms of stability, it has to be considered during formulation development that some lipids such as triglycerides tend to initially crystallize into an almost spherical, meta-stable *α*-polymorphic form [283]. During storage and time-dependently, the crystals rearrange and transform into a more ordered, platelet-like, and thermodynamically stable *β* form. Thus, this causes the formation of new surfaces, which could result in instabilities in the absence of a sufficient formulation, on the one hand [284], and impact the drug-loading capacity of particles, on the other hand. Previous studies indicated that a higher drug-loading capacity is achieved when the crystallinity of particles is lower and, thus, drug molecules could be embedded in the defects of the crystal lattice [285]. During particle recrystallization into the stable *β*-polymorphic form, the number of defects decrease, which can cause drug expulsion [286] and result in crystallization of the pure drug outside of the lipid particle. Thus, the general aim during formulation development should be to either stabilize the solid lipid nanoparticles directly in the *α*-polymorphic form to prevent polymorphic transition during storage or to formulate particles in the stable *β* form right at the beginning. Although particles in the *β*-polymorphic form cannot embed drug molecules in the crystal lattice, high drug loads can be realized with platelet-shaped (tri)glycerides particles (in the *β*-polymorphic form). The high surface-to-volume ratios enable the accommodation of drug molecules at particle surfaces, which results in high drug loads [283,287]. Investigations with amphotericin B and curcumin showed that, for these drugs, higher loads can be realized with solid lipid nanoparticles as carrier systems compared to lipid nanoemulsions, where embedding in a liquid matrix is possible [246].

Solid lipid nanoparticles have been investigated as carrier systems in various fields for application. Generally, similar to lipid nanoemulsions, they showed that they are biocompatible and suitable for improving the oral bioavailability of embedded substances. Furthermore, they proved to be advantageous in cancer therapy by overcoming several physiological barriers that usually obstruct the delivery of drugs to tumors [288]. Lately, solid lipid nanoparticles have also proved to be a delivery platform of interest for antibiotics, e.g., by overcoming some of the resistance mechanisms that bacterial strains have developed or by providing protection against enzymatic drug degradation [289].

Although prepared lipid nanosuspensions are liquid at the beginning, several studies have focused on further processing drug-loaded nanoparticles to semi-solid or solid dosage forms in order to improve their physical and chemical stability. Recently, freeze- and spray drying have often been used in combination with different matrix materials, such as lactose, glucose, mannitol, or trehalose, to prepare a stable solid formulation [264,290,291]. Studies embedding different lipids in an orodispersible film matrix indicated that this dosage form serves well as a delivery platform for lipid nanoparticles [67,292]. Furthermore, different mucoadhesive forms containing lipid nano- or microparticles were formulated, as follows: buccal films embedding didanosine [293], coumarin 6 [294], or fluconazole [295] into a lipid nanocarrier, as well as lyophilized sponges loaded with curcumin-containing lipid particles [296] or dextran hydrogels for the delivery of ibuprofen [297].

## 6. Major Findings and Future Perspectives

### 6.1. Major Findings

The variety of technologies in this review shows that different strategies are available depending on the drug substance, as well as the desired formulation properties. However, it should be noted that each formulation technology has both benefits and drawbacks and not all are suitable to formulate BCS class II and IV drugs. Table 2 provides an overview of the formulation technologies to facilitate selection.

The mentioned technologies are usually first used on a lab scale in a scientific environment. Their industrial relevance often goes hand in hand with their scalability to an industrial scale, but regulatory aspects must also be considered.

Drug nanoparticles have been produced by nanomilling in stirred media mills on an industrial scale for marketed products for more than two decades. The process parameterization for efficient and wear-avoiding process guidance is well established, while suitable formulations still mostly depend on experience and empirics. Accordingly, there are methods for formulation screening on the smallest of lab scales and for scaling up to production scale (such as in [27]). On the industrial scale, aseptic nanomilling lines are established. In contrast to these top-down processes, bottom-up processes, mainly continuous anti-solvent precipitation, are less popular for development and production beyond academic research.

ASDs are already produced on an industrial scale, and corresponding products are commercially available. Successful process development is ensured by using standard equipment (e.g., spray dryers, extruders, and electrospinners), which also allows for the application of established scaling-up strategies. In particular, manufacturing via hot-melt extrusion aligns with the trend toward establishing continuous production processes. Furthermore, this method eliminates the need for solvents. Considering FDM 3D printing as a manufacturing technology, scalability and regulatory issues are highly interconnected. FDM is primarily considered a point-of-care manufacturing technology, requiring centralized filament manufacturing and distribution to end users for final preparation. However, current legislation does not permit the manufacturing of drug-loaded half-products and their distribution to facilities without a manufacturing license, hindering the scalability of this technology. The regulatory framework has to be adapted to reflect such distributed manufacturing models. Additionally, methods where ASD formation may occur in situ during the final printing step at the point of care are associated with uncertain quality requirements. Conventional compounding technologies typically do not alter the solid state of APIs, resulting in a gap in established testing procedures. Therefore, the industrial relevance of filament extrusion coupled with FDM 3D printing is currently low and the future uncertain. In electrospinning, there are two main strategies equipment manufacturers use to upscale production. Multi-needle and needleless electrospinning combined with a continuously moving collector in a roll-to-roll system allow for high production capacities. Multi-needle systems consist of an array of needles, while needleless systems can vary significantly in their overall design. When it comes to the challenges of scaling-up, SES is by far the easiest, followed by EES. Different equipment designs have been proposed for MES upscaling; however, because of the thermal and rheological demands of the polymer melts, MES is rarely implemented on an industrial scale.

Liposomal formulations have been available on the market for nearly three decades; however, they continue to present significant challenges when transitioning processes from laboratory to production scales. During scale-up, it is imperative to implement measures that ensure batch-to-batch homogeneity, particularly in maintaining uniform particle size, encapsulation efficiency, and stability [298]. Although thin-film hydration is most commonly used, from a sustainability perspective, microfluidic technologies offer promising solutions for scalable production processes without the use of organic solvents. In contrast to the success of liposomes, MM formulations remain a relatively rare solubilization technology for commercial formulations, with only a few products available on the market. This is surprising, as the production technology is relatively simple, scalable, and sustainable. In general, regarding regulatory aspects, (phospho-)lipids are considered safe excipients, as well as for pediatric use, which could further promote the use of lipid-based formulations for drug solubilization in future product development.

Lipid nanoemulsions have also been prepared on an industrial scale for several decades in the form of parenteral nutrition by high-pressure homogenization, indicating that there is good certainty of action and that there will be no major regulatory challenges. Lipid nanosuspensions can also be prepared by high-pressure homogenization, but this process should then take place at temperatures above the melting temperature of the lipid. The high temperatures could lead to degradation of the drug, and lipid crystallization, formation of supercooled melts, and gelation phenomena also have be taken into account during preparation. For these reasons, promising approaches focusing on continuous, scalable emulsification processes are still being researched.

### 6.2. Future Prospective

In the future, research on drug nanoparticles will further focus on the modeling and prediction of formulation parameters, especially stabilization based on the physicochemical properties of the drug molecule to be processed. Additionally, development may focus on wear avoidance or wear separation from product streams, mitigating this major drawback of nanomilling approaches. For nanoparticle precipitation processes, further developments toward continuous process chains for the concentration of nanosuspensions and safe solvent recovery and recycling would be needed to make such processes industrially feasible.

In terms of ASD formulations, in the future, new theoretical modeling approaches are needed to predict drug-in-polymer solubility, ASD stability, and dissolution to reduce experiment efforts. Also, the development of new techniques that enable a better understanding of characteristic ASD behavior is needed. One novel technology is stimulated Raman-scattering microscopy coupled with sum frequency generation, revealing complex phenomena involving water ingress, swelling, drug dissolution, amorphous–amorphous and liquid–liquid phase separation and recrystallization in dissolving ASDs [299]. Models that describe the observed complex behavior need to be developed to predict the physical events during dissolution, and not only the dissolution rate.

Similar to ASD formulations, future research in the field of lipid carrier systems should also focus on predicting the solubility of drugs in different lipids in order to realize high drug loads. In addition, the focus should be on the next generation of solid lipid nanoparticles from polyglycerol esters of fatty acids to overcome stability challenges caused by recrystallization and polymorphism.

In recent years, artificial intelligence (AI) has become an increasingly powerful tool in various areas of research and is also being used to address issues in the field of formulation development. Different AI and machine learning models and tools have already been developed for pharmaceutical product development [300], and new ones are continually being introduced in the literature. Highly specialized platforms such as ‘FormulationAI’ [301] and ‘FormulationDT’ [302] have been developed to support data-driven formulation development, taking into account the solubility aspects of poorly water-soluble drugs. With ‘PharmSD’, a machine leaning platform was developed that focuses exclusively on solid dispersion formulations by predicting the physical stability, dissolution type, and dissolution rate of different drug–polymer combinations [303]. What all of these AI-based models have in common is that they enable the rapid evaluation of possible formulation strategies without time-consuming wet experiments [303]. They also aim to predict pharmacokinetic parameters, drug dosages, and routes of administration [300]. However, a current challenge is the lack of reliable negative examples for training models, as often only positive examples are available [304]. In addition, the reproducibility of the models could be difficult due to different operating environments [301]. It is generally assumed that the prediction accuracy of AI-based models and their importance in formulation development will continue to increase in the coming years.

## 7. Conclusions

Poorly water-soluble drugs continue to pose a challenge in the development of suitable formulations that ensure high bioavailabilities of these compounds after oral administration. This review provides an overview of the physical modification strategies discussed in the literature to overcome these limitations, focusing on formulations for peroral dosage forms and highlighting the advancements in this field over the past decade. The following three major formulation categories were identified and discussed in detail: drug nanoparticles, solid dispersions, and lipid-based formulations.

Drug nanoparticles can increase the dissolution rate of poorly soluble substances and are mainly prepared by nanomilling or precipitation. Since top-down technology has been the most common in the literature in recent years, different trends in milling equipment and the latest solution-based strategies to overcome challenges during the preparation of nanoparticles were considered. In addition, various studies were highlighted that deal with the further processing of nanoparticles into powders, such as spray- or freeze drying and fluidized bed granulation, as well as more innovative dosage forms like oral films.

With regard to solid dispersions, this section focused majorly on the formulation of ASDs. In addition to discussing spray drying for the preparation of ASDs, different novel HME approaches were presented. Besides current trends in the use of carbon dioxide for the preparation of HMEs, the extrusion-based 3D printing technology FDM was highlighted, which enables the preparation of personalized dosage forms. The latest research findings in the field of electrospinning were then discussed, focusing on the three most relevant technologies in recent years, namely, solution (SES), melt (MES), and emulsion electrospinning (EES). In addition to ASDs, the focus in this section was also on crystalline solid dispersions and amorphous deposition in mesoporous systems, which enable the embedding of drugs in inorganic or organic highly porous structures and, thus, improve bioavailability.

Another established concept for improving the solubility and bioavailability of lipophilic drugs is their formulation in lipid-based formulations. For this purpose, various carrier systems of various sizes have been developed, with (mixed) micelles and liposomes being representatives of the smallest size category. In these formulation concepts, poorly water-soluble drug molecules are incorporated into lipophilic domains, facilitating drug solubilization. Lipid nanoemulsions are larger carrier systems that are either directly dispersed in aqueous media or formulated as self-emulsifying systems that form emulsions upon contact with aqueous media. Solid lipid nanoparticles are based on a lipid that is solid at room temperature and are also used as a carrier system for lipophilic drugs. Recent studies showed successful further processing of lipid-based formulations into solid dosage forms.

Although it was shown that ‘brick-dust’-like molecules, whose solubility is limited mainly due to their solid-state properties that lead to a high melting point, are often formulated in drug nanoparticles or solid dispersions and drugs with high lipophilicity, referred to as ‘grease-ball’ molecules, are primarily formulated in lipid systems, this article also showed that there are many exceptions. Strategies such as drug milling are primarily suitable for many various types of poorly soluble drugs, and the structure of liposomes allows for loading of hydrophilic and lipophilic compounds, indicating that an appropriate formulation strategy is highly dependent on more drug characteristics than only its solubility in water. Therefore, an appropriate formulation strategy has to be chosen that depends on the drug to be applied and the specific requirements for each formulation, such as, for example, the intended drug dose to be administered to the patient. The recent developments highlighted in this review show that, despite numerous publications, there is still room for innovation and improvement in current formulation strategies, and the possibilities are only limited by the researcher’s imagination.

## Figures and Tables

**Table 1:** Advantages and disadvantages of solution (SES), melt (MES), and emulsion electrospinning (EES).

	Advantages	Disadvantages
**SES**	Processing of many polymers and drugs without restriction Simple equipment Adjustable release profiles (coaxial, triaxial, and side-by-side)	Loading capacity is limited by solubility Compatible solvent–drug combination is necessary Limited thickness of the fiber mat
**MES**	High drug-loading capacity No solvent usage or residue Wide temperature range Well-defined architecture via melt electrowriting (size, shape, and volume)	Heat-sensitive substances (e.g., proteins and nucleic acids) cannot be processed More complex device setup Limited output
**EES**	Encapsulation of hydrophilic drugs/bioactive agents in a hydrophobic polymer matrix Simple way to create core–shell structures Enhanced stability of drug Higher encapsulation efficiency	Difficult to scale-up Required surfactants can cause toxicity Stability problems during electrospinning

**Table 2:** Overview of the different technologies and their formulation principles, focusing on their potential application for poorly water-soluble BCS class II or IV drugs, as well as the benefits and drawbacks of the technologies.

Technology	Principle	BCS Class	Benefits	Drawbacks
Nanomilling of drug particles	Nanonization	II (IV)	Well-established technique Process scale-up possible Further processing into solid form established	Product contamination by grinding beads Nanoparticle agglomeration
Precipitation of nanoparticles	Nanonization	II (IV)	Continuous processing possible	Residues of solvents in product Low production rate Challenging scale-up Further processing to solid form
Spray drying with protein carriers	ASD formation	II	Improved stability and drug loading observed	New method, limited knowledge Low bulk density
HME	ASD formation	II	Well established Continuous process Solvent-free Extensive mixing	High processing temperatures Powder flowability and feedability can be limiting
HME with CO_2_	ASD formation	II	Continuous process Solvent-free Intensified mixing Moderate temperatures compared with conventional HME	New method, limited knowledge Powder flowability and feedability can be limiting
HME coupled FDM	ASD formation	II	In situ ASD formation possible Enables individualization Dissolution adjustment via geometric adjustments Point-of-care manufacturing possible	Second heating step Requires feedstock material Specific feedstock mechanical properties required
Electrospinning	ASD formation	II	Low thermal stress (EES and SES) High surface area Versatility in formulation (hydrophilic/hydrophobic drugs and polymers)	Scale-up limitations (esp., MES) High solvent consumption (EES) Solvent residue concerns (EES, SES) Process complexity
Mesoporous systems and aerogels	Drug amorphization	II (IV)	Stabilization of amorphous drugs Controlled and/or targeted drug release Dry and low-temperature loading processes possible	Carriers can negatively influence properties of, for example, tablets Physically limited loading capacity
Liposomes	Drug solubilization	II, IV	Controlled and/or targeted drug release Applicable for hydrophilic and hydrophobic drugs	Low loading capacity for lipophilic drugs High production cost
Mixed micelles	Drug solubilization	II, IV	High biocompatibility and low toxicity Simple production technology	Low drug-loading capacity Less suitable for large molecules
Lipid nanoemulsions	Dissolution in lipids	II, IV	Suitable for highly lipophilic drugs Positive impact of lipid Low thermal stress	Limited drug-loading capacity Unstable systems
Solid-lipid-based formulations	Dissolution in lipids	II, IV	Suitable for highly lipophilic drugs Positive impact of lipid	Low drug-loading capacity Lipids can undergo polymorphic transformations Preparation at high temperatures

## Data Availability

Data are contained within the article.
